# Feces production as a form of social immunity in an insect with facultative maternal care

**DOI:** 10.1186/s12862-015-0330-4

**Published:** 2015-03-12

**Authors:** Janina MC Diehl, Maximilian Körner, Michael Pietsch, Joël Meunier

**Affiliations:** Department of Evolutionary Biology, Institute of Zoology, Johannes Gutenberg University of Mainz, Mainz, Germany; Department of Hygiene and Environmental Medicine, Institute of Medical Microbiology and Hygiene, University of Mainz Medical Center, Mainz, Germany

**Keywords:** Social immunity, Family life, Feces, Precocial, Insect, Earwig

## Abstract

**Background:**

Social animals have the unique capability of mounting social defenses against pathogens. Over the last decades, social immunity has been extensively studied in species with obligatory and permanent forms of social life. However, its occurrence in less derived social systems and thus its role in the early evolution of group-living remains unclear. Here, we investigated whether lining nests with feces is a form of social immunity against microbial growth in the European earwig *Forficula auricularia*, an insect with temporary family life and facultative maternal care.

**Results:**

Using a total of 415 inhibition zone assays, we showed that earwig feces inhibit the growth of two GRAM+ bacteria, two fungi, but not of a GRAM- bacteria. These inhibitions did not result from the consumed food or the nesting environment. We then demonstrated that the antimicrobial activity against fungus was higher in offspring than maternal feces, but that this difference was absent against bacteria. Finally, we showed that family interactions inhibited the antibacterial activity of maternal feces against one of the two GRAM+ bacteria, whereas it had no effect on the one of nymphal feces. By contrast, antifungal activities of the feces were independent of mother-offspring interactions.

**Conclusion:**

These results demonstrate that social immunity occurs in a species with simple and facultative social life, and thus shed light on the general importance of this process in the evolution of group-living. These results also emphasize that defecation can be under selection for other life-history traits than simple waste disposal.

## Background

One of the major costs of group-living is its inherent risk of pathogen infection for group members [1-3]. While solitary species can only use personal immune responses to fight against infections, group-living species also possess the unique capability of mounting collective immune defenses, a phenomenon called social immunity [2,4]. Over the last two decades, a growing number of studies showed that multiple forms of social immunity can be expressed in species with permanent and obligatory social life, such as eusocial insects (reviewed in [2]). These studies were of great interest for the development of research on social immunity in insects, because they demonstrated that the high risks of pathogen infection associated with obligatory and complex forms of social life were likely to select for the emergence of collective defenses against pathogens [2,4]. However, they were of limited relevance to understand whether social immunity only emerged in eusocial systems and therefore represents a secondary trait derived from eusociality, or whether it also occurs in less derived forms of group-living and thus possibly plays a central role in the early evolution of group living organisms [2,4].

One method to address this issue is to investigate the occurrence of social immunity in species with temporary and facultative group-living. This is the case of species with family life, which represents a common form of group-living in insects [5,6], can be temporary and facultative such as in precocial species [7,8] and is generally considered as a major step in the evolutionary route to eusocial systems [6,9]. In insects, family life is broadly associated with the expression of care to the eggs and/or juveniles, such as protection against predators, clutch displacement and food provisioning [5,10]. Family life may also include forms of social immunity before egg hatching. For instance, parents groom their eggs to prevent the development of fungal spores in the European earwig *Forficula auricularia* [11], apply bacteria with antifungal properties to brood cell prior to oviposition in the European beewolf *Philanthus triangulum* [12], coat their nest with antimicrobial secretions in the housefly *Musca domestica* [13] or prophylactically avoid nest sites with high microbial pressure in the burying beetle *Nicrophorus vespilloides* [14]. Although pre-hatching forms of social immunity have been well studied in insects, surprisingly little is known about the nature and occurrence of the post-hatching ones (see e.g. in vertebrates [15,16]). Only recent studies showed that parental anal exudates and larval secretions exhibit antimicrobial properties in the burying beetles [17-19]. In this species, however, larvae feed on the carcass serving as nesting habitat, so that these antimicrobial mechanisms could also reflect evolutionary responses to competition with microbes over food access and/or to the extraordinarily high microbial pressure in this specific habitat.

In this study, we investigated whether social immunity occurs in the form of the production of feces with antimicrobial activity in the European earwig *F. auricularia*, an insect with temporary and facultative family life. In this species, mothers provide care to their offspring in soil burrows for several months, during which all family members - once hatched - line ground and walls with their feces pellets [8,20-23]. Earwig maternal care can take multiple forms, such as egg and juveniles (called nymphs) attendance and food provisioning through regurgitation, which have been shown to enhance offspring fitness [8,11,24,25]. Nevertheless, nymph survival does not require maternal care, as nymphs are mobile at hatching and can forage for themselves [8,26]. Here, we first tested whether (1) earwig feces provides a form of social immunity by inhibiting the development of bacteria and fungus into the nest, and determined whether these effects were independent of the consumed food and nesting material. We then investigated whether (2) antimicrobial activity was stronger in maternal compared to nymphal feces, as expected under the assumption that it reflects a post-hatching form of maternal care. Finally, we tested whether (3) the antimicrobial activity of feces is a socially-mediated trait that is triggered or inhibited by experiencing mother-offspring interactions [17]. If antimicrobial properties are induced by mother-offspring interactions, we predict that the feces produced by isolated individuals show lower antimicrobial activities. Conversely, we predict higher antimicrobial activities in feces produced by the isolated individuals if the costs of producing antimicrobial agents in the feces entail a mother-offspring conflict, in which each party tries to reduce its own investment into the production of antimicrobial components while benefiting from that of the other.

## Methods

### Insect rearing and feces collection

We collected feces pellets in 17 *F. auricularia* families composed of one mother and 36.11 ± 15.8 (mean ± SD) nymphs. These mothers were the first laboratory-born generation of individuals field sampled in 2012 in Dolcedo, Italy, and then maintained under standard laboratory conditions (rearing details in [27]). To determine whether the occurrence of mother-offspring interactions influences the antimicrobial properties of maternal and nymphal feces, the 17 families were randomly distributed among two groups at egg hatching. In the first group, we experimentally prevented mother-offspring interactions by separating mothers from their clutch of nymphs one day after egg hatching (Isolation group, n = 10). By contrast, mothers in the second group were separated from their nymphs ten days after egg hatching (Family group, n = 7). These separations were done by transferring the mother and the clutch of nymphs to two new petri dishes. At day 10, mothers and groups of nymphs from family groups were separated and transferred into two new petri dishes, in which they were maintained until feces collection at day 13 (first developmental instars). This manipulation was also done on the individuals from the isolation groups to standardize the experimental process. The transfer and three day delay between separation and feces collection ensured that the collected feces was relatively fresh and in large enough quantity to conduct the radial diffusion assays.

Individuals received *ad libitum* standardized food (for food composition, see [27]) from day 1 to day 9, and *ad libitum* green-colored pollen (Hochland Bio-Blütenpollen by Hoyer; Food die by DEKO BACK) from day 10 to day 12. Under these conditions, orphaning does not affect nymph quality in terms of developmental time and survival rate (Koch LK and Meunier J, unpublished data). The use of colored pollen is common in earwig experiments (e.g. [21,25,28]) and was used here to disentangle feces pellets from sand grains in the rearing containers. At day 13, all (colored) feces pellets present in each petri dish were collected using a sterile 10 μl pipette tip. For each petri dish, the total amount of collected pellets was weighed to the nearest 0.1 μg (Pescale), then suspended in 500 μl sterile NaCl solution (0.9%) and finally stored at 4°C. This feces solution was used 2.6 ± 1.5 days (mean ± SD) later to conduct the radial diffusion assays (see below). All petri dishes (diameters 10 and 5 cm before and after separation, respectively) contained humid sand as substrate and a plastic shelter as a nest. They were maintained in a climate chamber at 60% humidity, constant 20°C and 10:14 h light/dark cycle during the course of the experiment.

### Radial diffusion assays

We tested the antimicrobial properties of maternal and nymphal feces using a total of 170 radial diffusion assays against two GRAM+ bacteria, one GRAM- bacteria, and two fungi species (see details below). Radial diffusion assays were conducted in petri dishes (diameter 10 cm) filled with PDA (Potato Dextrose Agar, 70139, SIGMA-ALDRICH) covered with a solution of 10^9^ bacteria or spores/ml. Four samples were tested per plate. To this end, each fourth of a PDA plate received a blank disc (antimicrobial susceptibility test discs, OXOID) in its center, on which 10 μl of feces solution was preliminary applied. The same process was used to conduct a total of 245 controls (49 per microbial species), in which we tested whether growth inhibition could result from the NaCl solution used to dilute the feces (n = 15/species), the food eaten by the tested individuals (10 mg of colored pollen pellets suspended in 1 ml NaCl solution, n = 15/species; 240 mg of standardized food source suspended in 1 ml NaCl solution, n = 4/species) or the sand on which feces has been released (50 mg of sand suspended in 1 ml NaCl solution; n = 15/species). After inoculation, each plate was incubated at 36°C/24 h for bacteria and at 20°C/48 h or 20°C/72 h for the fungus (for *Saccharomyces cerevisiae* and *Aspergillus niger*, respectively). At the end of the incubation, the zone of clearance (diameter from one edge of the zone of inhibition to the other) was measured three times per sample and then averaged to give one mean value called antimicrobial activity.

The radial diffusion assays were conducted against five microbial species covering a spectrum of groups that have the capability to grow into earwig burrows. First, we used *Staphylococcus aureus* (NCIMB 9518), which is a GRAM+ bacteria known to secrete a range of enzymes and toxins associated with several diseases in vertebrates and invertebrates [29]. Second, *Bacillus subtilis* (ATCC 6633) is another GRAM+ bacteria, which is a facultative pathogen commonly found in the soil [29]. Third, *Escherichia coli* (ATCC 25922) is a GRAM- bacteria typically found in the intestinal tracts of mammals and insects [29]. Fourth, *Saccharomyces cerevisiae* (ATCC 2601) is a fungus known to cause lethal infections in invertebrates [29]. Finally, *Aspergillus niger* (wild type strain) is a fungus growing on rotten plant material that can be an opportunistic pathogen [29].

### Statistical analyses

We first tested the effect of feces producer (mother or nymphs), family life (isolation or family group) and their interaction on the log-transformed amount of feces produced between day 10 and 13 (i.e. the amount of diluted feces) using a linear model. Because inhibition zones do not follow normal distributions and include a substantial number of zeros across the radial diffusion assays (see results), the significance of the effects of feces producer and family life on antimicrobial activity were then tested using a series of randomized analysis of variance (randomized ANOVA; [30]). This non-parametric method allows estimating the significance of a factor (i.e. calculate p-values) by running a series of 10’000 ANOVA, in which the response variable (i.e. antimicrobial activity or antimicrobial activity per mg of feces) is permuted across the explanatory factors (i.e. feces producer and family life). Finally, we conducted pairwise comparisons between the antimicrobial activities of the controls (pooled) and the ones of the maternal or the nymphal feces using Mann–Whitney rank tests, in which the significance level α = 0.05 was adjusted for multiple testing to α = 0.025 using Bonferonni correction. All statistical analyses were conducted using the software R v3.1.1 (http://www.r-project.org). The R script to conduct randomized ANOVA is available on demand.

## Results

Each mother produced on average 13.06 ± 2.34 mg (mean ± SE) of feces between day 10 and day 13. This quantity was smaller than the 180.63 ± 18.88 mg of feces produced by the clutch of nymphs during the same period of time (Likelihood Ratio (LR) χ^2^_1_ = 252.72, P < 0.0001). The total amount of feces produced over three days was independent of family isolation (LR χ^2^_1_ = 0.97, P = 0.324), or of an interaction between family isolation and feces producer (LR χ^2^_1_ = 1.12, P = 0.290).

**Figure 1:**
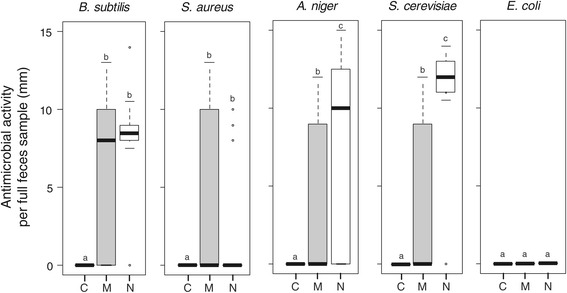
**Antimicrobial activities of controls (C), maternal (M, grey) and nymphal (N, white) feces.** Controls combine assays with NaCl, Pollen, Food and Sand. Boxplots depict median (bold bar) and interquartile range (light bar), with outlying values (circles) and whiskers extending to 1.5 times the interquartile range. Different letters indicate P < 0.005.

Inhibition zones were found in 25 (73.5%) assays against *B. subtilis*, 10 (29.4%) against *S. aureus*, 19 (55.9%) against *S. cerevisiae*, 17 (50.0%) against *A. niger*, but none (0.0%) against *E. coli.* Maternal feces inhibited the growth of at least one microbial species in 13 (76.5%) of the 17 tested families, while nymphal feces had inhibition effects in every sample from the 17 (100%) families. None of the controls (NaCl, pollen, standardized food and sand) showed antimicrobial activity in any of the 245 assays (Figure 1).

The antimicrobial activity of maternal and nymphal feces produced over three days depended on the feces producer and the microbial species, but not on the occurrence of mother-offspring interactions (Table 1a, Figure 1). Specifically, antimicrobial activities against *A. niger and S. cerevisae* were lower in maternal compared to nymphal feces, whereas antimicrobial activities against *B. subtilis* and *S. aureus* were independent of feces producer (Table 1a). Except against *E. coli*, each type of feces showed higher antimicrobial activity than the controls (Table 2, Figure 1). The general antibacterial activity of nymphal feces against *S. aureus* was mostly driven by three points in the data set (Figure 1). If these three points were excluded, the resulting mean antibacterial activity of nymphal feces against *S. aureus* would become null and thus smaller than the one of maternal feces (Mann–Whitney test, W = 168, p = 0.008) and similar to the controls (Figure 1).

**Table 1:** **Influences of feces producer and mother-offspring interactions on antimicrobial activities (a) per full sample and (b) per mg of feces**

	***B. subtilis***	***S. aureus***	***A. niger***	***S. cerevisiae***
*(a) Activity per full feces sample*				
Feces producer (FP)	P = 0.440	P = 0.098	**P = 0.005**	**P <0.0001**
Mother-offspring interactions (MO)	P = 0.808	P = 0.104	P = 0.342	P = 0.051
FP : MO	P = 0.553	P = 0.934	P = 0.068	P = 0.215
*(b) Activity per mg of feces*				
Feces producer (FP)	**P <0.0001**	**P = 0.008**	P = 0.095	P = 0.080
Mother-offspring interactions (MO)	P = 0.813	P = 0.575	P = 0.052	P = 0.078
FP : MO	P = 0.812	P = 0.731	**P = 0.037**	P = 0.089

Feces producers were either the mother or the nymphs. P-values were obtained from randomized ANOVAs and the significant ones are in bold.

**Table 2:** **Comparisons between inhibition zones generated by the controls and the total amount of either maternal or nymphal feces**

	***B. subtilis***		***S. aureus***		***A. niger***		***S. cerevisiae***	
	***W***	***P***	***W***	***P***	***W***	***P***	***W***	***P***
Maternal feces	686	**<0.0001**	588	**<0.0001**	539	**<0.0001**	539	**<0.0001**
Nymphal feces	759.5	**<0.0001**	490	**0.0028**	710.5	**<0.0001**	759.5	**<0.0001**

Statistical values were obtained from Mann–Whitney tests. Significant P-values are in bold. All p-values remain significant after correcting for multiple testing.

**Figure 2:**
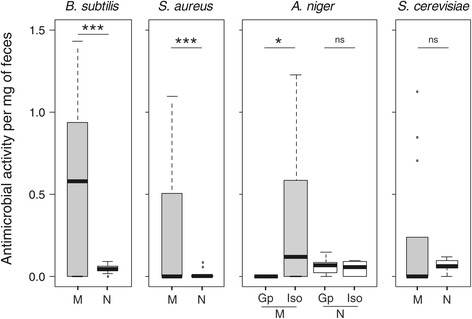
**Antimicrobial activities per mg of maternal (M, grey) and nymphal (N, white) feces.** When reported, feces producers were either maintained in family groups (Gp) or isolated (Iso) before feces collection. Boxplots depict median (bold bar) and interquartile range (light bar), with outlying values (circles) and whiskers extending to 1.5 times the interquartile range. ***P < 0.001; *P < 0.05; ^ns^P > 0.05.

In line with the prediction that antifungal components are more concentrated in maternal than nymphal feces, we found that the antimicrobial activities per mg of feces against *B. subtilis and S. aureus* were larger in maternal compared to nymphal feces (Table 1b, Figure 2). By contrast, feces producer did not influence such activity against *S. cerevisae* (Table 1b, Figure 2). Overall, the occurrence of mother-offspring interactions did not shape the antimicrobial activities per mg of feces against *B. subtilis*, *S. aureus* and *S. cerevisae* (Table 1b, Figure 2). However, it interacted with feces producer to shape the antimicrobial activity per mg of feces against *A. niger* (Table 1b, Figure 2). Specifically, the presence of mother-offspring interactions canceled the antimicrobial activity of maternal feces (Mann–Whitney rank test; W = 52.5, P = 0.040) but had no effect on the one of nymphal feces (Figure 2, W = 38, P = 0.807). Note that this interaction was only marginally non-significant when analyzing the overall antimicrobial activity of maternal feces produced over three days (Table 1a).

There was no family effect on the antimicrobial activities of nymphal and maternal feces (Table 3). Across microbial species, antimicrobial activities were comparable (present or absent) between maternal and nymphal feces in 51.4% of the families, a value that was not significantly different from a random distribution (Binomial test against 50%, P = 0.904). Note that the four microbial species (excluding *E. coli*) did not influence the proportion of families with comparable antimicrobial activities between maternal and nymphal feces (i.e. both present plus both absent *versus* present in only one type; Pearson’s Chi-squares test, *χ*^2^ = 3.0, df = 3, P = 0.391).

**Table 3:** **Expression of feces antimicrobial activity per family**

**Antimicrobial activity in**		***B. subtilis***	***S. aureus***	***A. niger***	***S. cerevisiae***	***E. coli***
**Maternal feces**	**Nymphal feces**					
Yes	Yes	8	2	3	5	0
No	No	0	9	3	3	17
Yes	No	3	5	2	0	0
No	Yes	6	1	9	9	0

For each of the five microbial species, we reported the number of family in which an antimicrobial activity was found in both maternal and nymphal feces, in none of them or in either maternal or nymphal feces.

## Discussion

Gaining a better understanding of the evolution of the multiple forms of group-living requires insights into the mechanisms that help individuals to limit the inherent risk of infection. Here, we demonstrate that lining nests with feces inhibits microbial development in the European earwig. Specifically, earwig feces showed antimicrobial activities against two GRAM+ bacteria (*B. subtilis* and *S. aureus*) and two fungi (*A. niger* and *S. cerevisiae*). These antimicrobial properties are likely to provide immune benefits to earwig family members, as many microbial entomopathogens have the capability to grow under the underground conditions provided by insect nests (e.g. [1,31]), several of them are known to frequently attack earwig nests [32-35], and a recent study showed that even the development of non-entomopathogenic fungus into the nest comes with detrimental effects on earwig fitness [11]. Together with the fact that earwig nymphs produce more feces when encountering related compared to unrelated conspecific juveniles [21], these results thus support that feces production at least partly reflects a kin-triggered form of social immunity.

The maintenance of feces in the nest is a poorly studied phenomenon in eusocial insects [36,37], in which colony members are generally assumed to evacuate feces into specific nest chambers to prevent microbial development in the colony (reviewed in [2,38]). This phenomenon has nevertheless been reported in two non-eusocial insects exclusively feeding on their nesting material, the wood cockroach *Cryptocercus punctulatus* and the burying beetle *N. vespilloides* [14,19,39], for which the use of anal exudates (and their antimicrobial activity) into the nest has been proposed to have at least partially evolved to limit competition with microbes over food access [19,40].

Our study shows that the total amount of feces produced by mothers over three days did not exhibit higher antimicrobial activities than the one produced by nymphs, revealing that feces antimicrobial activity is not a simple form of post-hatching maternal care. Instead, we show that nymphs contributed more to antifungal nest protection than mothers, mostly due to their overall larger production of feces (each mg of nymphal feces exhibited similar antifungal activity than each mg of maternal feces). This higher feces production also allowed nymphs to compensate for the lower intrinsic antibacterial activity of their feces (activity per mg of feces) against GRAM+ bacteria, thus exhibiting an overall antimicrobial activity comparable to the one of maternal feces. This age-specific effect on the antimicrobial activity per mg of feces suggests differences in composition between nymphal and maternal feces. Feces compositions could differ in terms of quantity and/or quality of residual compounds of their personal immunity, which are known to be present in the feces and to become stronger with aging in other insect species [41-45]. Another discrepancy in feces composition could result from differing hindgut flora of mothers and nymphs. The insects gut includes a great variety of symbiotic microorganisms that are crucial for growth and protection against infections [46-48], but that also change with aging [43,49,50]. Finally, nymphal and maternal feces could vary in terms of chemical products released during defecation. For instance, earwigs possess a pygidial gland on their abdomen that releases chemicals with antimicrobial properties [51]. Disentangling among these three non-mutually exclusive hypotheses will be addressed in further studies by investigating the presence of immune components and antimicrobial chemicals inside the feces, as well as by characterizing earwig gut flora.

We found that the antimicrobial activity of maternal feces depended on preliminary interactions with their nymphs. Specifically, family interactions inhibited the antimicrobial activity of maternal feces against *S. cerevisiae*, whereas they had no effect on the one of nymphal feces. This latter result contrasts with the one found in the burying beetle *N. vespilloides*, in which the absence of tending parents lowered the level of antibacterial activity in larvae exudates [17]. In earwigs, our result first reveals that the presence and/or quantity of the compounds mediating the antimicrobial activity of maternal feces against *S. cerevisiae* are socially-dependent. More generally, it suggests that mothers can adapt their investment into such a form of social immunity to the investment expressed by their nymphs. Assuming that investment into social immunity is energetically costly (see e.g. [52]), such maternal strategy could be adaptive and allow mothers to re-allocate their energy into other important life-history traits, such as forms of care and future reproduction [8,27]. Nevertheless, the effect of family life on feces antimicrobial activities was absent with the four other tested microbial species, indicating that the compounds mediating this activity are fixed during the period of family life. These compounds do not come from the environment, as there was no antimicrobial activity in the food consumed by the individuals and in the sand covering the rearing containers.

A somewhat surprising result of our study was the large number of feces samples with no antimicrobial activity. These negative assays are unlikely to reflect a problem in our methodology, as radial diffusion assay is a standard procedure that has been commonly used to test antimicrobial activities in other insect species (e.g. [18,36]). They are also unlikely to reflect that feces antimicrobial activity is a family-trait only expressed in a limited number of families, since we showed that the occurrence (or absence) of feces antimicrobial activities was not necessarily the same between nymphs and mothers from the same family. Conversely, our result could reflect a form of specificity in the immune responses mediated by the feces, which is in line with the fact that almost every feces sample inhibited the growth of at least one of the tested microbes. Another explanation could be that feces producers need some cues to switch on antimicrobial activity in their feces. These cues are unlikely to come from our standardized rearing environment, but might reflect that some field sampled mothers have been naturally exposed to pathogens prior sampling, and that such exposure affected the immunity of their own descendants through transgenerational immune priming [53]. However, the occurrence of transgenerational immune priming remains to be tested in *F. auricularia*.

Although earwig feces showed antimicrobial activity against the two tested GRAM+ bacteria, this activity was absent against the GRAM- bacteria *E. coli*. This lack of activity against *E. coli* has been reported in the antimicrobial secretions of other insect, such as the burying beetle *N. vespilloides* [18]. It may reflect either (1) higher physiological costs of mounting antimicrobial protection against GRAM- bacteria [41], (2) low selection pressure to mount defenses against GRAM- bacteria, e.g. because they are not present in their natural habitat or are important symbiotic organisms in the gut flora (but see [49]), or (3) specific resistance of the tested bacterial strain against the antimicrobial compounds present in earwig feces. Further studies should address this issue.

## Conclusion

Overall, we demonstrate that social immunity in the form of lining nest with antimicrobial compounds can emerge and persist in species with primitive forms of group-living. Mounting collective defenses against microbial development could therefore be a widespread phenomenon across social systems and an important one in the early evolution of social life, as it does not require that individuals live in permanent and obligatory groups, and/or that group members compete with microbes for access to nest material as a food source. Interestingly, these results also emphasize that defecation does not only reflect individual needs of waste disposal, but can be under selection for its importance in other crucial life-history traits [19,38,39].

## Availability of supporting data

The data set supporting the results of this article is available in the DRYAD repository, http://doi:10.5061/dryad.9p31r [54].

## References

[1] Schmid-Hempel P (1998). Parasites in Social Insects.

[2] Cremer S, Armitage SAO, Schmid-Hempel P (2007). Social immunity. Curr Biol.

[3] Masri L, Cremer S (2014). Individual and social immunisation in insects. Trends Immunol.

[4] Cotter SC, Kilner RM (2010). Personal immunity versus social immunity. Behav Ecol.

[5] Wong JWY, Meunier J, Kölliker M (2013). The evolution of parental care in insects: the roles of ecology, life history and the social environment. Ecol Entomol.

[6] Royle NJ, Smiseth PT, Kölliker M (2012). The Evolution of Parental Care.

[7] Smiseth PT, Darwell CT, Moore AJ (2003). Partial begging: an empirical model for the early evolution of offspring signalling. Proc R Soc B Biol Sci.

[8] Kölliker M (2007). Benefits and costs of earwig (*Forficula auricularia*) family life. Behav Ecol Sociobiol.

[9] Bourke AFG, Franks NR (1995). Social Evolution in Ants.

[10] Trumbo ST, Royle NJ, Smiseth PT, Kölliker M (2012). Patterns of parental care in invertebrates. Evol Parent care.

[11] Boos S, Meunier J, Pichon S, Kolliker M (2014). Maternal care provides antifungal protection to eggs in the European earwig. Behav Ecol.

[12] Kaltenpoth M, Göttler W, Herzner G, Strohm E (2005). Symbiotic bacteria protect wasp larvae from fungal infestation. Curr Biol.

[13] Cardoza YJ, Klepzig KD, Raffa KF (2006). Bacteria in oral secretions of an endophytic insect inhibit antagonistic fungi. Ecol Entomol.

[14] Rozen DE, Engelmoer DJP, Smiseth PT (2008). Antimicrobial strategies in burying beetles breeding on carrion. Proc Natl Acad Sci U S A.

[15] Gasparini J, Mccoy KD, Staszewski V, Haussy C (2006). Dynamics of anti-Borrelia antibodies in Black- legged Kittiwake (*Rissa tridactyla*) chicks suggest a maternal educational effect. Can J Zool.

[16] Jacquin L, Blottière L, Haussy C, Perret S, Gasparini J (2012). Prenatal and postnatal parental effects on immunity and growth in “lactating” pigeons. Funct Ecol.

[17] Reavey CE, Beare L, Cotter SC (2014). Parental care influences social immunity in burying beetle larvae. Ecol Entomol.

[18] Arce AN, Smiseth PT, Rozen DE (2013). Antimicrobial secretions and social immunity in larval burying beetles, *Nicrophorus vespilloides*. Anim Behav.

[19] Cotter SC, Kilner RM (2010). Sexual division of antibacterial resource defence in breeding burying beetles, *Nicrophorus vespilloides*. J Anim Ecol.

[20] Costa JT (2006). Dermaptera. Earwig mothers. Other insect Soc.

[21] Falk J, Wong JWY, Kölliker M, Meunier J (2014). Sibling cooperation in earwig families provides insights into the early evolution of social life. Am Nat.

[22] Koch LK, Meunier J (2014). Mother and offspring fitness in an insect with maternal care: phenotypic trade-offs between egg number, egg mass and egg care. BMC Evol Biol.

[23] Wong JWY, Meunier J, Lucas C, Kölliker M (2014). Paternal signature in kin recognition cues of a social insect: concealed in juveniles, revealed in adults. Proc R Soc B Biol Sci.

[24] Meunier J, Kölliker M (2013). Inbreeding depression in an insect with maternal care: influences of family interactions, life stage and offspring sex. J Evol Biol.

[25] Staerkle M, Kölliker M (2008). Maternal food regurgitation to nymphs in earwigs (*Forficula auricularia*). Ethology.

[26] Meunier J, Kölliker M (2012). When it is costly to have a caring mother: food limitation erases the benefits of parental care in earwigs. Biol Lett.

[27] Meunier J, Wong JWY, Gómez Y, Kuttler S, Röllin L, Stucki D (2012). One clutch or two clutches? Fitness correlates of coexisting alternative female life-histories in the European earwig. Evol Ecol.

[28] Meunier J, Kölliker M (2012). Parental antagonism and parent-offspring co-adaptation interact to shape family life. Proc R Soc B Biol Sci.

[29] Kayser FH, Bienz KA, Eckert J, Zinkernagel RM (1998). Medizinische Mikrobiologie.

[30] Manly BFJ (1997). Randomization, Bootstrap and Monte Carlo Methods in Biology.

[31] Reber A, Chapuisat M (2011). Diversity, prevalence and virulence of fungal entomopathogens in colonies of the ant Formica selysi. Insectes Soc.

[32] Miller JS, Zink AG (2012). Parental care trade-offs and the role of filial cannibalism in the maritime earwig, *Anisolabis maritima*. Anim Behav.

[33] Ben-Ze’ev IS (1986). Notes on Entomophthorales (Zygomycotina) collected by T. Petch: II. Erynia ellisiana sp. nov., non Erynia forficulae (Giard.), comb. nov., pathogens of Forficulidae (Dermaptera). Mycotaxon.

[34] Hodson AK, Friedman ML, Wu LN, Lewis EE (2011). European earwig (*Forficula auricularia*) as a novel host for the entomopathogenic nematode Steinernema carpocapsae. J Invertebr Pathol.

[35] Wang W, LU W, Li Z (1994). *Furia shandongensis* (Zygomycetes: Entomophthorales), a new pathogen of earwigs. Mycotaxon.

[36] Rosengaus RB, Guldin MR, Traniello JFA (1998). Inhibitory effect of termite fecal pellets on fungal spore germination. J Chem Ecol.

[37] Chouvenc T, Efstathion CA, Elliott ML, Su N (2013). Extended disease resistance emerging from the faecal nest of a subterranean termite. Proc R Soc B Biol Sci.

[38] Weiss MR (2006). Defecation behavior and ecology of insects. Annu Rev Entomol.

[39] Rosengaus RB, Mead K, Du Comb WS, Benson RW, Godoy VG (2013). Nest sanitation through defecation: antifungal properties of wood cockroach feces. Naturwissenschaften.

[40] Otti O, Tragust S, Feldhaar H (2014). Unifying external and internal immune defences. Trends Ecol Evol.

[41] Beckage NE (2008). Insect Immunology.

[42] Shao Q, Yang B, Xu Q, Li X, Lu Z, Wang C (2012). Hindgut innate immunity and regulation of fecal microbiota through melanization in insects. J Biol Chem.

[43] Dillon RJ, Dillon VM (2004). The gut bacteria of insects: nonpathogenic interactions. Annu Rev Entomol.

[44] Schmid-Hempel P (2005). Evolutionary ecology of insect immune defenses. Annu Rev Entomol.

[45] Zerofsky M, Harel E, Silverman N, Tatar M (2005). Aging of the innate immune response in *Drosophila melanogaster*. Aging Cell.

[46] Engel P, Moran NA (2013). The gut microbiota of insects - diversity in structure and function. FEMS Microbiol Rev.

[47] Kaltenpoth M, Steiger S (2014). Unearthing carrion beetles’ microbiome: characterization of bacterial and fungal hindgut communities across the Silphidae. Mol Ecol.

[48] Kaltenpoth M, Engl T (2014). Defensive microbial symbionts in Hymenoptera. Funct Ecol.

[49] Kaltenpoth M (2009). Actinobacteria as mutualists: general healthcare for insects?. Trends Microbiol.

[50] Dillon RJ, Webster G, Weightman AJ, Keith Charnley A (2010). Diversity of gut microbiota increases with aging and starvation in the desert locust. Antonie Van Leeuwenhoek.

[51] Gasch T, Schott M, Wehrenfennig C, Düring R-A, Vilcinskas A (2013). Multifunctional weaponry: the chemical defenses or earwigs. J Insect Physiol.

[52] Cotter SC, Topham E, Price AJP, Kilner RM (2010). Fitness costs associated with mounting a social immune response. Ecol Lett.

[53] Moret Y (2006). “Trans-generational immune priming”: specific enhancement of the antimicrobial immune response in the mealworm beetle, *Tenebrio molitor*. Proc R Soc B Biol Sci.

[54] Diehl JMC, Körner M, Pietsch M, Meunier J. Feces production as a form of social immunity in an insect with facultative maternal care. Dryad. http://doi:10.5061/dryad.9p31r 10.1186/s12862-015-0330-4 PMC4408575 25888183

